# Validation of a New Measuring Instrument for the Assessment of Bite Force

**DOI:** 10.3390/diagnostics13233498

**Published:** 2023-11-21

**Authors:** Ina Nitschke, Celine Moede, Werner Hopfenmüller, Bernhard A. J. Sobotta, Andreas Koenig, Julia Jockusch

**Affiliations:** 1 Gerodontology Section, Department of Prosthetic Dentistry and Materials Science, Leipzig University, Liebigstraße 12, 04103 Leipzig, Germany or ina.nitschke@zzm.uzh.ch (I.N.); celine.moede@medizin.uni-leipzig.de (C.M.); bernhard.sobotta@medizin.uni-leipzig.de (B.A.J.S.); 2 Clinic of General, Special Care and Geriatric Dentistry, Center of Dental Medicine, University of Zurich, Plattenstrasse 11, CH-8032 Zurich, Switzerland; 3 Institute of Biometry and Clinical Epidemiology, Charité-Universitätsmedizin Berlin, Corporate Member of Freie Universität Berlin, Humboldt-Universität zu Berlin, and Berlin Institute of Health, 10117 Berlin, Germany; werner.hopfenmueller@charite.de; 4 Materials Science Section, Department of Prosthetic Dentistry and Materials Science, Leipzig University, Liebigstraße 12, 04103 Leipzig, Germany; 5 University Research Priority Program “Dynamics of Healthy Aging”, University of Zurich, CH-8050 Zurich, Switzerland

**Keywords:** occlusal force, measuring device, prototype, measurement accuracy, chewing function

## Abstract

Background: this study aims to validate two occlusal-force-measuring devices by comparing them to a universal testing machine and assessing their reliability across various dental and prosthetic groups. The research comprised two parts: part 1 assessed the measurement accuracy of the Occlusal Force Meter GM 10^®^ (OFM) (Morita, Nagano Keiki, Higashimagome, Ohta-ku, Tokyo, Japan) and a prototype (PRO) by comparing them to a calibrated universal testing machine (ZWICK). Part 2 involved analyzing the devices’ reliability based on clinical bite force measurements from study participants. Results: both devices become more accurate and reliable compared to the ZWICK over time of usage. Additionally, higher deviation from the ZWICK can be observed for higher values of forces applied and vice versa for both devices. The PRO’s intraoral alignment influences its mean values compared to the OFM in different dental and prosthetic groups. Conclusion: both devices had limitations and required quadratic function calibration, making them suitable only for progression measurements. The study concludes that both the OFM and PRO devices can measure occlusal forces with improved accuracy over time. Intraoral alignment should be considered. Their easy-to-use clinical application would allow a more widespread use of masticatory function diagnosis, which could indicate the need for treatment and improve treatment planning.

## 1. Introduction

The function of the masticatory system is described by the parameters occlusal force (also bite force, masticatory force, and occlusal force) and chewing efficiency. While chewing efficiency is the objectively measurable chewing performance that can be reproducibly evaluated by tests [1,2,3,4,5,6,7,8,9,10,11], occlusal force is the physiologically applied force when comminuting a piece of food.

What is the relevance of occlusal force measurements for clinical practice and research? It is known that the quality of life is influenced by the function of the masticatory system [12]. Any direct reported impact on oral health by a patient is a patient-reported outcome. It can be measured in four dimensions (oral function, orofacial appearance, pain, and psychosocial impact) with patient-reported outcome measurements (PROM) such as the Oral Health Impact Profile (OHIP) [13,14,15]. An impaired chewing function with pain or reduced bite force and chewing efficiency may be components of deteriorating oral function and one reason why patients seek contact with a dentist. Dental treatment focuses on the aspects of esthetics, speech, and function. It seems surprising that it is assumed that restoration of lost tooth structure by means of removable dentures or fixed partial restorations, for example, is generally equated to the restoration of masticatory function. In daily dental practice, this assumption is never objectively checked. In other words, chewing function is not objectively diagnosed before treatment planning and any possible functional change is not assessed after treatment has been completed.

High or low values of occlusal forces alone have no direct significance for oral function. They are only part of the factors to be assessed that make up chewing function as part of oral function. Several factors may influence this function of the masticatory system, e.g., a reduced number of teeth or occlusal surfaces, age [16], number of antagonistic teeth contacts [17], saliva flow rate, lip function, cheek, tongue, soft palate [18], and the quality and the type of the denture [19] or cognitive state [20].

The significance of high or low values of measured occlusal forces in relation to oral function may depend on several factors, and it is important to consider both the clinical and individual context. High occlusal forces may indicate strong masticatory muscles and a good ability to chew and process food effectively. This is generally considered a positive sign of oral function. However, excessive occlusal forces, especially if they are not evenly distributed across the dental arches, can lead to problems such as tooth wear, fractures, and temporomandibular joint (TMD) problems. These problems can have a detrimental effect on oral function and overall oral health. Additionally, patients with TMD tend to show lower occlusal forces than their healthy counterparts [21]. Low occlusal forces can be beneficial for people with sensitive teeth or dentures. They can reduce the risk of dental trauma and tooth wear. However, extremely low occlusal forces may indicate muscle weakness or dysfunction, which can lead to difficulty chewing, affect food intake, and may lead to malnutrition. The even distribution of occlusal forces is a key factor in oral function. An imbalance of occlusal forces can lead to problems such as misaligned teeth and difficulty biting, chewing, and speaking. It is important to know that there are significant individual differences in occlusal forces. What is high or low for one person may be normal for another. Therefore, clinical assessment and comparison to baseline or reference values for the individual are critical in determining significance.

In contrast to daily dental practice, many clinical studies assess both occlusal force (e.g., [22,23,24]) and chewing efficiency (e.g., [25,26,27]) within a wide variety of populations. A variety of measurement methods and instruments are used for this purpose.

Differing values for occlusal force have been reported in the literature, depending on the anatomical and physiological characteristics of the subjects, as well as other factors, such as their aging process [28]. Another reason why there may be different values for occlusal force [21,29] is that many measuring methods and devices differ greatly in their design, in their method of measurement, and in their use of these measuring instruments: they differ in application or the position of alignment and in the number of measurements, as well as their localization (uni- vs. bilateral) or influencing factors such as the necessary mouth opening when performing the measurement [30]. Various devices have been used for the direct measurement of occlusal forces. These include bite forks, strain gauges, quartz and foil transducers, pressure rubber tubing, gnathodynamometer, pressure sensitive foil, and force transducers [28].

A frequently used method is the measurement of occlusal force with the Occlusal Force Meter GM 10^®^ (OFM) (Morita, Nagano Keiki, Higashimagome, Ohta-ku, Tokyo, Japan). Its accuracy and repeatability have been proven in previous studies [31,32,33,34,35,36,37,38]. The method allows simple, fast, and reproducible measurement of occlusal force. The measurement is performed in the region of the first molar by biting with the maximum possible jaw-closing force. The occlusal force meter is no longer manufactured. Moreover, the instrument has not been available to researchers or dentists in the European region. At the same time, however, interest in clinical research on chewing function is increasing strongly. This has resulted in the need to develop and validate a new measuring device for the evaluation of occlusal force. For this reason, a German company developed a prototype for occlusal force measurement (PRO) for scientific surveys and for practicing dentists to measure occlusal force in everyday clinical practice.

The aim of the present study is (a) to assess the reliability of the OFM and the PRO for occlusal force measurement by means of a comparative measurement with a standardized calibrated universal testing machine (ZWICK) and (b) to prove the validity and reliability of the prototype PRO compared with the OFM in a clinical study.

The authors hypothesize that (a) the measured values of the OFM and PRO for occlusal force will not differ from each other but that (b) there will be differences when compared with the values of a standardized, calibrated universal testing machine (ZWICK).

## 2. Materials and Methods

### 2.1. Study Design

The study was approved by the competent ethics committee of the University of Leipzig (number: 048/21-ek, approval date 8 October 2021). All participants or their legal representatives gave written informed consent.

The study was divided into two parts:

- Part 1: Measurement accuracy: verification of the reliability of the values measured with the two occlusal force measuring devices in comparison with a standardized calibrated universal testing machine (ZWICK);
- Part 2: Reliability analysis of the two occlusal force measuring devices in a clinical study.

### 2.2. Study Population

Participants were only required in study part 2 to measure occlusal forces in a clinical setting.

Participants 18 years of age or older were recruited for this study. An upper age limit was not set. Participants should not be experiencing any acute problems such as pain or abscesses that would require emergency treatment. Furthermore, participants should not suffer from a known craniomandibular dysfunction, or one diagnosed at the beginning of the study. There should be at least one antagonistic contact per jaw side including prosthetic restoration, and no non-occlusion in the posterior region. Individuals with paralysis or neuralgia in the oral and facial region, congenital mental retardation (e.g., Down syndrome or cerebral palsy), or acquired cognitive impairment (e.g., dementia) were excluded from participation.

Since the number of teeth [17,39] and the type of prosthetic restoration [19] are assumed to have an effect on occlusal force, the participants were divided into five groups according to their dental and prosthetic status as modified according to the description in the 5th German Oral Health Study (5. Deutsche Mundgesundheitsstudie, DMS V) [39,40]. These leading groups were group 1—fully dentate (natural dentition, no or fixed dentures), group 2—partially dentate without dentures, group 3—partially dentate with removable partial dentures in at least one jaw, group 4—edentulous with complete dentures in both jaws and two interforaminal implants in the lower jaw, and group 5—edentulous with complete dentures in at least one jaw. The lowest-value restoration in one of the two jaws is the basis for assigning participants to a specific group. This means that a participant who had a full dentition in the upper jaw and a complete denture in the lower jaw was assigned to the leading group, “edentulous with complete dentures”.

### 2.3. Study Measuring Devices and Their Application

**Figure 1:**
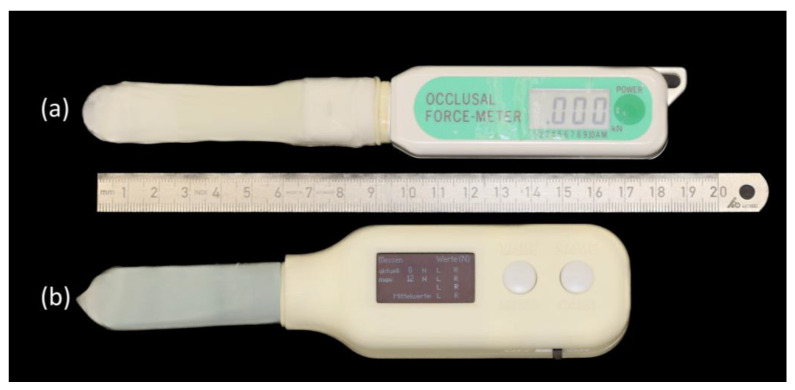
(**a**) Occlusal Force Meter GM 10 for measuring the occlusal force in kilo Newtons (kN). (**b**) Prototype of the Bredent medical Company for measuring the occlusal force in Newtons (N).

In the study, the occlusal force was measured using the Occlusal Force Meter GM 10^®^ (Morita, Nagano Keiki, Higashimagome, Ohta-ku, Tokyo, Japan) (referred to as OFM) (Figure 1a) and the prototype for measuring the occlusal force from Bredent medical GmbH & Co. KG (Senden, Germany) (referred to as PRO) (Figure 1b). Both gauges are portable hydraulic compression force gauges. The weight of the OFM is 63.52 g (grams); that of the PRO, 93.89 g.

The OFM has been used in many studies [24,41,42,43]. It has a biting element made of a vinyl material enclosed in a polyethylene tube surrounded by a plastic cover. This makes it hard, stiff, and difficult to bite. Measurements were carried out according to the procedure described in the literature [44] and the manufacturer’s instructions. Due to the construction of the device, the measurement with the OFM was performed in transversal (buccal–oral) alignment in the region of the first molar or the closest area (dentures inserted, if available) on the right or left side of the jaw (OFM_right_ and OFM_left_, respectively). The participants were asked to apply the maximum possible jaw-closing force.

The PRO is a 3D-printed plastic device with a glycerin-gel-filled measuring finger that measures the voltage change (volts, V) and translates it into the corresponding occlusal force (Newtons, N). The measured value is then shown on a digital display. The device can temporarily store three measured values per measuring point and determine the average value from these. The measuring finger is encased in a plastically deformable plastic, resulting in a bite block of 10 mm. Due to the construction of the device, the measurements were carried out at different interocclusal positions on both sides of the jaw: alignment of the measuring finger in (1) sagittal alignment (anterior–posterior) (PRO_sag_right_; PRO_sag_left_), (2) transversal alignment (buccal–oral) in the region of the premolars (PRO_trans_PM_right_; PRO_trans_PM_left_), and (3) transversal alignment (buccal–oral) in the region of the molars (PRO_trans_M_right_; PRO_trans_M_left_) (Table 1).

### 2.4. Measurement Procedures

- (a) With the universal testing machine (ZWICK).

**Figure 2:**
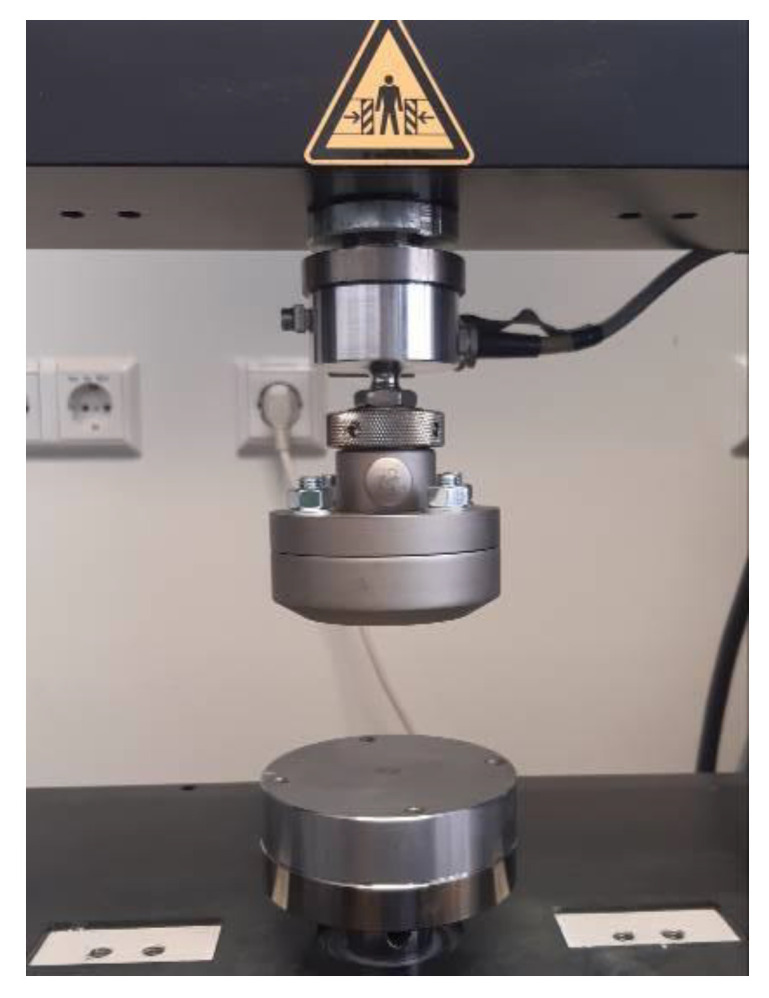
Experimental setup in the universal testing machine “ZWICK” (Retroline Z010 ZwickRoell, Ulm, Germany) for uniaxial compressive measurements.

To check the accuracy of the two measuring instruments, control measurements were carried out at intervals of approx. 500 measurements with each device. A standardized universal testing machine (Retroline Z010, ZwickRoell, Ulm, Germany) (referred to as ZWICK in the following) was used for this purpose. Its measurements were defined as the reference. Both measuring devices were positioned in the same way in the ZWICK (Figure 2).

To check the reliability of the theoretical occlusal force values measured by the OFM and the PRO, a comparison with the universal testing machine (ZWICK) as the reference was carried out. For this purpose, forces between 0 and 700 N were applied by the ZWICK to both devices according to the manufacturer’s specifications of both devices.

The theoretical occlusal force values measured by the OFM and PRO were displayed graphically, and their deviations from the target value (force applied by the universal testing machine (ZWICK) in Newtons at a loading speed of 0.3 mm/min (other loading speeds, e.g., 0.6 and 0.9 mm/min, produced similar results)) were calculated as polynomial coefficients.

These polynomial coefficients were then used to convert the measured theoretical occlusal forces into effective occlusal forces for both OFM and PRO.

The re-checking procedure was carried out at several time points (T1–T5) over a period of 12 months when the two devices, the OFM and the PRO, were used to measure the occlusal force of participants in a clinical setting (see above). This resulted in five polynomial coefficients with which the measured theoretical occlusal forces of the OFM and the PRO were corrected into effective occlusal forces accordingly. Through this procedure, it was possible to calculate and visualize the deviation from the set point (applied forces of the ZWICK) of the two devices.

- (b) In participants.

The occlusal force measurements for each participant were taken at one appointment. The PRO measuring device was always used first, followed by the OFM measuring device. At each of the eight measuring sites (Table 1), three measurements—applying the maximum occlusal force of the participant—were recorded, and the mean values were calculated. These measured values are referred to as theoretical occlusal forces in this paper.

### 2.5. Statistical Objective

Since this is an exploratory pilot study and estimates are missing regarding the primary endpoints, no power analysis was performed. The present study targeted 30 participants per leading group (five groups based on dental and prosthetic status; 150 participants in total) for an initial evaluation.

In study part 1, graphical visualization of validity and reliability was performed using the OFM and PRO versus the ZWICK machine (reference) at five different time points (T1–T5). Linear coefficients for each device at each time point were calculated as described before, and the effective occlusal forces were calculated.

In study part 2, the first step was to adjust the collected measured theoretical occlusal forces of OFM and PRO according to the measurement time point using the linear coefficient determined in study part 1 (calculation of effective occlusal forces). A *t*-test with a significance level at α < 0.05 was conducted. Significant values indicate that the mean values of the two measuring devices (OFM and PRO) differ from each other. Here, the corresponding measuring region of the PRO device should not be used for the evaluation of the occlusal force.

Afterward, Bland–Altman plots were used as a graphical visualization method for the comparison of the two measuring methods (OFM/PRO). For this purpose, the OFM was set as the gold standard. In each plot, the upper and lower limits were set using the 1.96-fold median values of the reference instrument (OFM) separately for each of the five lead groups.

Reliability analysis was run to calculate intraclass correlation coefficients (ICC). ICC estimates and their 95% confidence intervals (CI) were calculated using SPSS statistical package version 27. The interpretation of the ICCs was based on the recommendation of Koo and Li (2016) [45]. ICC values less than 0.5 are indicative of poor reliability, values between 0.5 and 0.75 indicate moderate reliability, values between 0.75 and 0.9 indicate good reliability, and values greater than 0.90 indicate excellent reliability. For the interpretation, the ICC itself and the confidence intervals were used.

## 3. Results

### 3.1. Part 1: Measurement Accuracy: Verification of the Reliability of the Measured Values of the Two Occlusal Force Measuring Devices in Comparison to a Standardized Testing Machine

**Figure 3:**
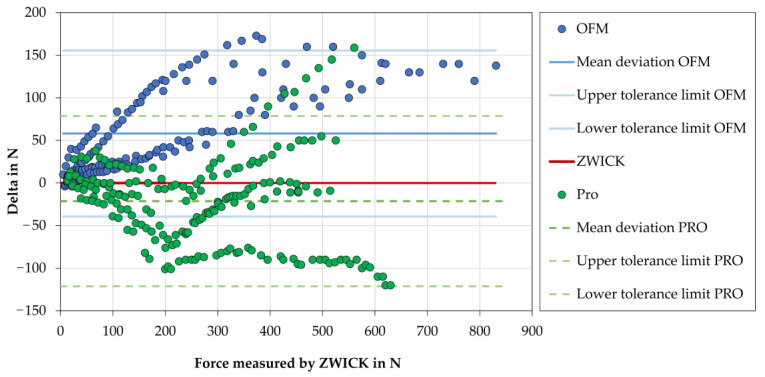
Graphical representation of the forces applied by the ZWICK testing machine (loading speed 0.3 mm/min) in Newtons (N) (set point) and the difference of the forces measured (theoretical occlusal forces) by the two occlusal force devices (OFM—occlusal force meter, PRO—prototype) in Newtons (N) compared to the ZWICK.

The theoretical occlusal forces indicated by the OFM and the PRO at a force applied by the ZWICK machine in Newtons (load speed 0.3 mm/min) were systematically collected and plotted (Figure 3). The minimum and maximum deviations (in percent, %) from the set point of the ZWICK testing machine are tabulated in Table 2.

The OFM indicates a lower force at all five measuring time points than the force applied by the ZWICK (set point). The OFM showed very similar and constant measured values at time points T3, T4, and T5. The PRO runs below the set point at T1, T2, and T4, consequently showing a greater force than was output by the ZWICK. At T2 and T4, the displayed values of the PRO approach are very close to the set point from 300 N onward. At time T3, the values displayed by the PRO almost correspond to the set point, and from 350 N, lower values are displayed. At time T5, the theoretical occlusal force values of the PRO run above the set point and, therefore, display lower values. The values displayed by the PRO at this time are very similar to the values displayed by the OFM. Overall, with the increase in measurement duration, both devices (OFM and PRO) became more and more accurate and reliable compared to the gold standard (ZWICK).

### 3.2. Part 2: Comparison of Reliability Analysis of the Two Occlusal Force Measuring Devices

Clinical measurements of occlusal forces were performed on the participants in the five groups based on dental and prosthetic status. The socio-demographic characteristics of the subjects as a whole and separately by leading group are described in Table 3.

The corrected values (effective occlusal forces) measured with the two devices by the leading group are shown in Appendix A (Table A1). It was shown that the intraoral alignment of the PRO does seem to have an influence on its mean values when compared to the mean values of the OFM in the five different leading groups. For fully dentate participants with no or fixed dentures and for partially dentate participants without dentures, the sagittal intraoral alignment of the PRO should be favored on the right side of the jaw. In contrast, an intraoral transversal alignment of the PRO in the premolar or molar region should be used for partially dentate participants with removable dentures. For edentulous participants with complete dentures, edentulous participants with complete dentures and implant support in the lower jaw, and measurements of fully dentate or partially dentate participants on the left side of the jaw, none of the alignments of the PRO resulted in identical values as measured with the OFM. Here, the values of the PRO were significantly lower (edentulous participants with and without implant support) or higher (fully or partially dentate participants), respectively, than those of the OFM for each alignment of the PRO (Appendix A, Table A1).

A graphical comparison of the two measuring devices was undertaken with Bland–Altman plots by the measuring region (direction of alignment of the PRO) (Table 4).

The reliability analysis of the occlusal-force-measuring devices showed that there is mostly a moderate agreement between the PRO and the OFM for most measurements. The best agreement was achieved in the “partially dentate, removable denture” group (good to excellent agreement) (Table 4) (Appendix A, Table A2).

In the “fully dentate, no or fixed denture” group, a tendency toward transversal intraoral alignment of the PRO in the premolar region appeared. The “partially dentate, no denture” group, the “partially dentate, removable denture” group, and the “edentulous, complete denture with implant support” group showed no influence on the intraoral alignment of the PRO. The “edentulous, complete denture” group showed a tendency toward conformity of the PRO with the OFM for transversal intraoral alignment of the PRO in the molar region (ICC moderate to good) (Table 4) (Appendix A, Table A2).

## 4. Discussion

Based on the results of the study, the authors must reject their null hypothesis that the measured values of the two tested devices do not differ from each other. Furthermore, the hypothesis can be accepted that there are differences in the measured values of the two devices, OFM and PRO, compared to the ZWICK machine.

### 4.1. Principles of Bite Force Measurement

The assessment of oral function is an important task in clinical dentistry and research, and measuring bite force is a relevant and objective way since it correlates with masticatory performance [46]. However, there are limitations to many of the bite-force-measuring devices that have been developed in the past. This could also be the reason why the measurement of chewing force and chewing efficacy has not yet found its way into everyday dental routine diagnostics. This lack could partly be explained by technical challenges to accurately measuring bite force and by the absence of a generally accepted methodology and equipment.

One approach to bite force measurements uses strain gauge transducers. However, such devices are very sensitive to temperature and humidity. The space required for isolation and the use of thermocouples for internal calibration are at odds with the limited space available in the mouth and can interfere with normal occlusion [38]. Piezoresistive and pressure transducers, on the other hand, lack accuracy and reliability [33]. Additionally, piezoelectric transducers are believed to be insufficiently sensitive [33], and some devices, such as the dental prescale system, are not widely available outside of Japan and require specialized analytical equipment for data analysis [47].

Some low-cost sensors that are available, such as the one developed by Fastier-Wooller et al. (2016), present limitations due to their thickness [48]. Other options may not work in edentulous patients or are still in an experimental stage or suffer from low reliability [49,50,51].

Newer diagnostic methods, such as the bionic jaw motion system, make it possible to precisely examine the function of the masticatory system under static and dynamic conditions [52]. The bite forces measured in the clinical context are important for the settings used to record and reproduce a realistic jaw relationship.

### 4.2. Comparison of Bite Force Values

In the “fully dentate” group, the mean bite force values for the occlusal force measurement (OFM) ranged between 457.9 ± 235.7 N and 485.0 ± 243.4 N, while for the portable recording occlusal (PRO) device, the values varied from 555.2 ± 423.8 N to 782.1 ± 565.0 N, contingent upon the device alignment.

These findings were similar in range to those reported in the literature. For instance, Abu Alhaija et al. (2009) demonstrated that the mean biting force (MBF) among adults from Jordan averaged 573.42 ± 140.18 N. Individuals with a shorter facial structure exhibited the highest MBF (679.60 ± 117.46 N), while those with longer facial types displayed the lowest MBF (453.57 ± 98.30 N). Additionally, the MBF averaged 599.02 ± 145.91 N for males and 546.97 ± 131.18 N for females [42].

Braun et al. (1995) reported a mean maximum bite force of 738 ± 209 N among dentate subjects [53]. Al-Zarea (2015) indicated that the mean maximum bite force was 596.2 ± 76.3 N on the natural tooth side and 580.9 ± 74.3 N on the fixed partial denture side [41]. Gibbs et al. (2002) evaluated mean values of 720 N (range: 244 to 1243 N) for fully dentate participants aged 18 to 55 years. This study showed lower median values for OFM, namely 493.2 N (range: 46 to 958 N) and 504.4 N (range: 39 to 975 N), and for PRO, ranging from 396.2 N (range: 12 to 3174 N) to 565.0 N (range: 8 to 3641 N) [54].

Sano and Shiga (2021) demonstrated masticatory forces in fully dentate subjects ranging from 416.4 N ± 103.7 N to 611.2 N ± 202.9 N, dependent on age and gender [22].

In the “partially dentate, no dentures” group, Gibbs et al. (2002) evaluated a mean bite force of 462 N (range: 98 to 1031 N) for participants aged 28 to 76 with posterior tooth loss, which is comparatively higher than the median masticatory force measured in this study at 231.8 N (range: 15 to 887 N) up to 238.0 N (range: 27 to 782 N) for the OFM. In comparison to PRO, similar values ranging from 336.3 N (range: 0 to 1172 N) to a maximum of 508.0 N (range: 107 to 957 N) were obtained, consistent with the findings of Gibbs et al. (2002) [54].

For edentulous individuals with complete dentures, Rismanchian et al. (2009) demonstrated masticatory forces of 5.65 kgf ± 1.46 kgf (kgf—kilogram-force, an obsolete physical unit for force, equivalent to 55.42 N ± 24.32 N) and 7.01 kgf ± 2.1 kgf (equivalent to 68.74 N ± 20.59 N), dependent on wear time [55]. These values are lower than the data collected for the edentulous group with complete dentures by the OFM but close to the value collected with the PRO in this study.

### 4.3. Study Limitations

#### 4.3.1. Prototype Design

In this study, two occlusal-force-measuring devices (OFM and PRO) were compared to a calibrated universal testing machine (ZWICK) in terms of reliability and comparability. The results show that both devices indicated different force values compared to the ZWICK. The OFM with its hard occlusal surface consistently showed lower values than those applied by the ZWICK. This observation is consistent with the results of the study by Serra and Manns (2013) [56]. Here, it was shown that measuring devices using a soft occlusal surface measured higher force values than devices with hard occlusal surfaces. The prototype (PRO) with its soft surface confirmed this finding. Compared with the ZWICK, the PRO even measured higher values than were actually applied [56].

It also appeared to the investigators that the thickness of the measuring finger was crucial in the design. The thicker finger of the PRO resulted in some prosthesis wearers being unable to transmit any force at all or only a limited force to the measuring finger.

#### 4.3.2. Quadratic Function Calibration

With the help of linear functions, it was possible to calculate the deviation from the set point (applied forces of the ZWICK) of the two devices. In this way, an “actual force” was calculated that must have acted on the masticatory force gauge to display the corresponding measured value. Using the “actual forces”, it is easier to compare the two gauges, as the deviations of the gauges from the nominal value no longer need to be taken into account. However, this possibility of calibration does not exist in the clinical setting. In clinical dentistry, the measuring devices should be suitable for analysis before and after prosthetic therapies, as well as for monitoring patients over time. Absolute measurements cannot be achieved with either of the measuring devices used in the study. Both only offer the possibility for progression measurements to quantitatively record changes before and after therapy or over time, as is the case with other measuring devices [57]. Other studies also used linear equations to calculate or convert the measured masticatory force for measuring devices in order to increase the value for clinical applications [58].

#### 4.3.3. Study Population and Measuring Devices

It is important to note that the present study has another limitation. The sample size was limited, especially in some groups with only a few participants. In addition, only certain types of dentures were included, while others such as implant-supported dentures or fixed dentures were not.

## 5. Conclusions

Overall, the results of this study show that both the OFM and the PRO are suitable for estimating occlusal forces at your level. The accuracy of the measurements increased with the duration of use for both devices. The results presented give an indication of the deviations to be expected with the devices. However, it is important to consider the intraoral alignment of the PRO to obtain accurate readings. Additionally, the variability of the two devices found in the study should be considered by others when using the devices. Estimation of bite forces with the devices is possible, but measurement requires calibrating to correct the values.

These results are important for the clinical application of occlusal-force-measuring devices. They can contribute to a better understanding of the patient’s reported oral health outcome and chewing function and the diagnosis of any chewing function impairment. Improved understanding of diagnostic data, in turn, may lead to improved treatment planning and, ultimately, clinical therapeutic outcomes for the patient.

## Figures and Tables

**Table 1:** Visualization of the localizations of the measuring points of the Occlusal Force Meter GM 10^®^ (OFM) and the prototype measuring device (PRO).

Device	Alignment of the Device in Specific Regions of the Jaw	Side of the Jaw	
		Right	Left
OFM	Transversal, molar regions	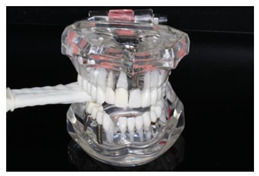	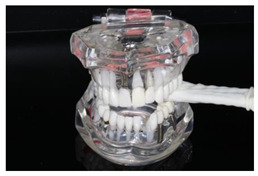
PRO	Sagittal	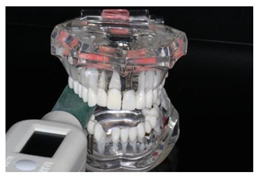	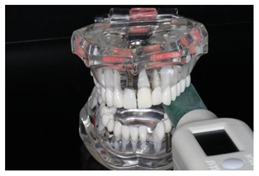
	Transversal, premolar regions	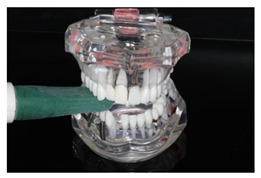	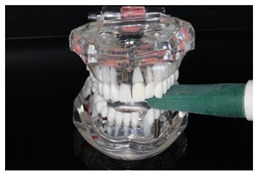
	Transversal, molar regions	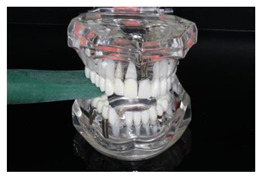	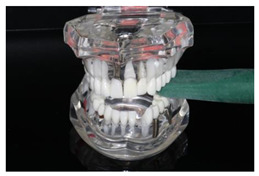

**Table 2:** The minimum and maximum deviations (in percent, %) from the set point (forces applied by the ZWICK testing machine (loading speed 0.3 mm/min) in Newtons (N)) measured per measurement time point (T1 to T5) and device (OFM—occlusal force meter, PRO—prototype), and the mean of the deviations. Negative values indicate higher values displayed by the OFM or PRO compared to the set point of the ZWICK testing machine.

Time Points	T1		T2		T3		T4		T5	
Devices	OFM	PRO	OFM	PRO	OFM	PRO	OFM	PRO	OFM	PRO
Deviation (%)										
Minimal	−78	−40	−50	−0.5	−23	−14	−35	−52	−47	−70
Maximal	−14	71	−36	60	61	13	−15	47	−17	3.3
Mean	−42	30	−45	19	−11	−3	−22	8	−32	−33

**Table 3:** Socio-demographic characteristics of the participants by leading group (fully dentate, no or fixed dentures; partially dentate, no denture; partially dentate, removable denture; edentulous with complete dentures and implant support in lower jaw, edentulous with complete dentures) according to the dental and prosthetic status modified in accordance with the 5th German Oral Health Study (DMS V) [39,40] and in total (years, SD—standard deviation).

	Fully Dentate	Partially Dentate		Edentulous		Total
	No/Fixed Denture	No Denture	Removable Denture	Complete Denture with Implant Support	Complete Denture	All Groups
	n = 61	n = 37	n = 46	n = 12	n = 42	n = 198
Sex (n/%)						
Male	26/42.6	25/67.6	21/45.7	4/33.3	22/52.4	98/49.5
Female	35/57.4	12/32.4	25/54.3	8/66.7	20/47.6	100/50.5
Age (years)						
Mean ± SD	50.3 ± 25.6	70.2 ± 14.2	72.7 ± 10.5	81 ± 8.5	73.7 ± 14.1	66.1 ± 20.6
Median (Range)	55.0 (20–89)	71.0 (33–93)	75.0 (46–90)	81.5 (69–95)	77.5 (34–94)	72.0 (20–95)

**Table 4:** Intraclass coefficient (ICC) and 95% confidence interval (CI) and its interpretation according to Koo and Li (2016) * [45] for each leading group (fully dentate, no or fixed dentures; partially dentate, no denture; partially dentate, removable denture; edentulous with complete dentures and implant support in the lower jaw, and edentulous with complete dentures) by the region of alignment of the prototype occlusal-force-measuring device (occlusal force meter (OFM) alignment in the molar region of the jaw right and left; prototype (PRO) alignment of the measuring finger in sagittal alignment (PRO_sag_), transversal alignment (buccal–oral) in the region of the premolars (PRO_trans_PM_), and transversal alignment (buccal–oral) in the region of the molars (PRO_trans_M_).

			∆PROsag		∆PROtrans_PM		∆PROtrans_M	
			Right 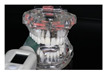	Left 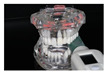	Right 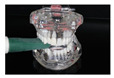	Left 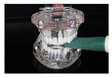	Right 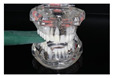	Left 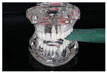
Fully dentate	No/fixed denture	ICC	0.444	0.397	0.594	0.507	0.545	0.293
		Min/Max	0.089/0.663	0.025/0.632	0.329/0.756	0.181/0.704	0.249/0.725	−0.114/0.561
Partially dentate	No denture	ICC	0.688	0.593	0.608	0.522	0.556	0.323
		Min/Max	0.365/0.848	0.169/0.803	0.159/0.813	−0.027/0.777	0.059/0.794	−0.292/0.689
	Removable denture	ICC	0.892	0.889	0.887	0.841	0.868	0.861
		Min/Max	0.792/0.943	0.740/0.947	0.793/0.939	0.708/0.913	0.750/0.930	0.741/0.926
Edentolous	Complete denture with implant	ICC	0.382	0.583	0.428	0.653	0.433	0.626
		Min/Max	−0.144/0.795	−0.228/0.890	−0.176/0.821	−0.230/0.909	−0.144/0.826	−0.263/0.899
	Complete denture	ICC	0.650	0.729	0.693	0.733	0.740	0.809
		Min/Max	0.268/0.824	0.434/0.864	0.325/0.850	0.482/0.860	0.414/0.875	0.533/0.912
**Legend**								
Interpretation of reliability *								
Excellent		>0.90						
Good		0.75–0.90						
Moderate		0.50–0.75						
Poor		<0.50						

## Data Availability

The data presented in this study are available on request from the corresponding author. The data are not publicly available due to privacy.

## Appendix A

**Table A1:** Effective occlusal force values in Newtons (N) separated by the intraoral alignment of the two devices (OFM—occlusal force meter; PRO—prototype) in specific regions of the jaw and separated by five leading groups modified based on dental and prosthetic status as described in the 5th German Oral Health Study (DMS V) [39,40]. The test statistic (*t*-test between OFM and PRO) shows the significance values p. The significance level was set at α < 0.05. If p values are significant (bold values), the mean values of the two measuring devices (OFM and PRO) differ from each other. In the case of significant differences between OFM and PRO, the corresponding measuring region of the PRO device should not be used for the evaluation of the occlusal force.

(a) Right Side of the Jaw									
			OFM	PRO					
			Right	Sag_Right		Trans_PM_Right		Trans_PM_Right	

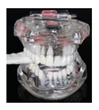
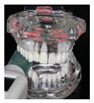
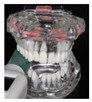
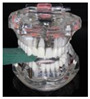
						
**Fully** **dentate**	No/fixed denture	Number	n = 61	n = 61	*p=* 0.066	n = 61	*p=* 0.143	n = 61	***p=*** **0.007**
		Mean ± SD	485 ± 243	620 ± 624		555 ± 424		640 ± 507	
		Median (Range)	504 (39–975)	396 (12–3174)		488 (20–1849)		480 (20–2407)	
**Partially dentate**	No denture	Number	n = 33	n = 31	*p=* 0.081	n = 33	***p=*** **0.001**	n = 27	***p=*** **0.006**
		Mean ± SD	265 ± 198	346 ± 281		425.2 ± 309.5		530 ± 490	
		Median (Range)	232 (15-887)	336 (0–1172		426.6 (0–1161)		437 (0–2522)	
	Removable denture	Number	n = 45	n = 43	***p=*** **0.025**	n = 44	*p=* 0.985	n = 40	*p=* 0.709
		Mean ± SD	182 ± 146	155 ± 175		186 ± 187		185 ± 246	
		Median (Range)	136 (11-640)	98 (14–888)		149 (4-833)		114 (3–1106)	
**Eden-tulous**	Complete dentures & implant support	Number	n = 12	n = 12	***p*<** **0.001**	n = 12	***p*<** **0.001**	n = 12	***p*<** **0.001**
		Mean ± SD	103 ± 47	29 ± 26		38 ± 25		31 ± 32	
		Median (Range)	92 (40–188)	19 (3–96)		34 (3-76)		17 (0–110)	
	Complete denture	Number	n = 42	n = 41	***p*<** **0.001**	n = 41	***p*<** **0.001**	n = 40	***p*<** **0.001**
		Mean ± SD	97 ± 104	49 ± 56		49 ± 62		56 ± 63	
		Median (Range)	69 (0-461)	32 (0-183)		22 (0–212)		38 (0–263)	
**(b) Left Side of the Jaw**									
			**OFM**	**PRO**					
			**Left**	**Sag_Left**		**Trans_PM_Left**		**Trans_PM_Left**	

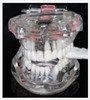
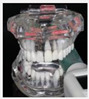
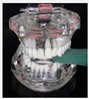
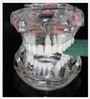
									
**Fully** **dentate**	No/fixed denture	Number	n = 61	n = 61	***p=*** **0.012**	n = 61	***p*<** **0.001**	n = 60	***p=*** **0.001**
		Mean ± SD	458 ± 236	698 ± 816		667 ± 511		782 ± 565	
		Median (Range)	493 (46–958)	427 (4–4005)		530 (40–2444)		565 (8–3641)	
**Partially dentate**	No denture	Number	n = 31	n = 31	***p=*** **0.017**	n = 3	***p*<** **0.001**	n = 21	***p=*** **0.002**
		Mean ± SD	265 ± 178	370 ± 286		435 ± 264		510 ± 235	
		Median (Range)	238 (27–782)	349 (0–1073)		404 (0–952)		508 (107–957)	
	Removable denture	Number	n = 45	n = 43	***p*<** **0.001**	n = 44	*p=* 0.854	n = 43	*p=* 0.372
		Mean ± SD	173 ± 165	134 ± 124		185 ± 144		165 ± 177	
		Median (Range)	121 (20–642)	86 (4–560)		149 (4–605)		87 (4–814)	
**Eden-tulous**	Complete denture & implant support	Number	n = 12	n = 12	***p*<** **0.001**	n = 12	***p=*** **0.002**	n = 12	***p*<** **0.001**
		Mean ± SD	110 ± 54	49 ± 39		63 ± 50		59 ± 47	
		Median (Range)	107 (32–200)	44 (0–119)		54 (12–188)		47 (8–171)	
	Complete denture	Number	n = 41	n = 41	***p=*** **0.002**	n = 42	***p=*** **0.009**	n = 39	***p*<** **0.001**
		Mean ± SD	91 ± 92	53 ± 62		67 ± 84		66 ± 77	
		Median (Range)	73 (0–428)	36 (0–220)		28 (0–313)		40 (0–308)	

**Table A2:** Bland–Altman plots for each leading group (fully dentate, no or fixed dentures; partially dentate, no denture; partially dentate, removable denture; edentulous with complete dentures and implant support in the lower jaw, and edentulous with complete dentures) by the region of alignment of the prototype occlusal force measuring device (occlusal force meter (OFM) alignment in the molar region of the jaw right and left: OFM_right_, OFM_left_; prototype (PRO) alignment of the measuring finger in sagittal alignment (PRO_sag_right_; PRO_sag_left_), transversal alignment (buccal–oral) in the region of the premolars (PRO_trans_PM_right_; PRO_trans_PM_left_), and transversal alignment (buccal–oral) in the region of the molars (PRO_trans_M_right_; PRO_trans_M_left_). Intraclass coefficient (ICC) and 95% confidence interval (CI) and its interpretation according to Koo and Li (2016) * [45].

**(a) Fully Dentate, No or Fixed Denture**			
Right side of the jaw	**ICC (95% CI)**	Left side of the jaw	**ICC (95% CI)**
[N] [N]	0.444 (0.089–0.663) interpretation of reliability *: poor (poor to moderate)	[N] [N]	0.397 (0.025–0.632) interpretation of reliability *: poor (poor to moderate)
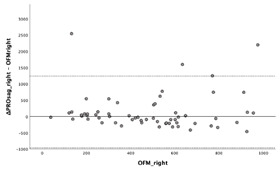
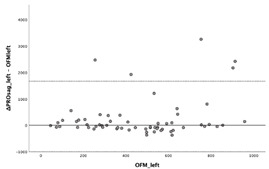
[N] [N]	0.594 (0.329–0.756) interpretation of reliability *: moderate (poor to good)	[N] [N]	0.507 (0.181–0.704) interpretation of reliability *: moderate (poor to moderate)
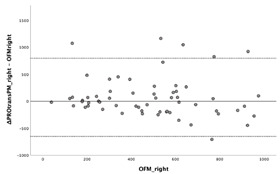
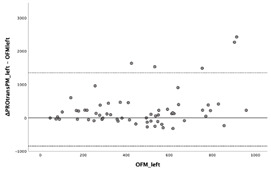
[N] [N]	0.545 (0.249–0.725) interpretation of reliability *: moderate (poor to moderate)	[N] [N]	0.293 (−0.114–0.561) interpretation of reliability *: poor (poor to moderate)
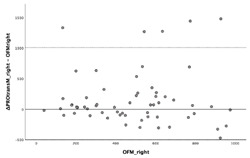
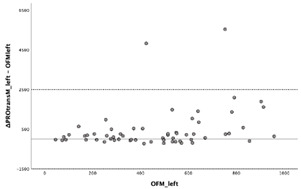
**(b) Partially Dentate, No Denture**			
Right side of the jaw	**ICC (95% CI)**	Left side of the jaw	**ICC (95% CI)**
[N] [N]	0.688 (0.365–0.848) interpretation of reliability *: moderate (poor to good)	[N] [N]	0.593 (0.169–0.803) interpretation of reliability *: moderate (poor to good)
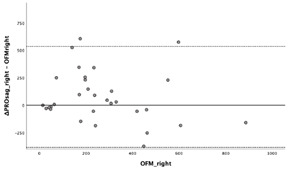
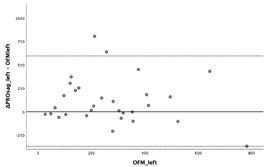
[N] [N]	0.608 (0.159–0.813) interpretation of reliability *: moderate (poor to good)	[N] [N]	0.522 (−0.027–0.777) interpretation of reliability *: moderate (poor to good)
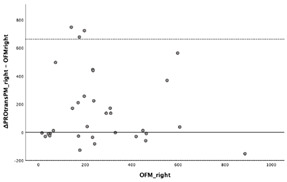
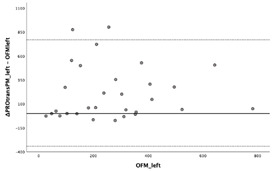
[N] [N]	0.556 (0.059–0.794) interpretation of reliability *: moderate (poor to good)	[N] [N]	0.323 (−0.292–0.689) interpretation of reliability *: poor (poor to moderate)
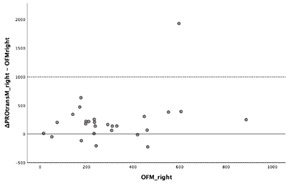
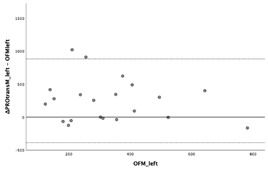
**(c) Partially Dentate, Removable Denture**			
Right side of the jaw	**ICC (95% CI)**	Left side of the jaw	**ICC (95% CI)**
[N] [N]	0.892 (0.792–0.943) interpretation of reliability *: good (good to excellent)	[N] [N]	0.889 (0.740–0.947) interpretation of reliability *: good (moderate to excellent)
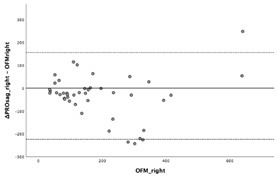
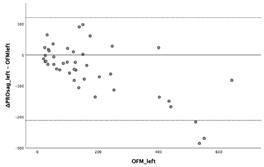
[N] [N]	0.887 (0.793–0.939) interpretation of reliability *: good (good to excellent)	[N] [N]	0.841 (0.708–0.913) interpretation of reliability *: good (moderate to excellent)
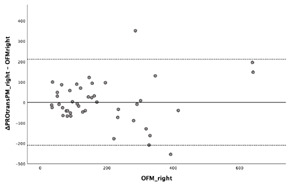
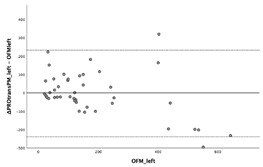
[N] [N]	0.868 (0.750–0.930) interpretation of reliability *: good (good to excellent)	[N] [N]	0.861 (0.741–0.926) interpretation of reliability *: good (moderate to excellent)
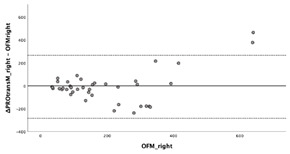
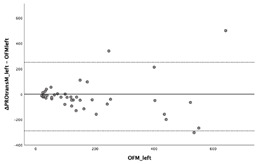
**(d) Edentulous, Complete Denture with Impant Support in Lower Jaw**			
Right side of the jaw	**ICC (95% CI)**	Left side of the jaw	**ICC (95% CI)**
[N] [N]	0.382 (−0.144–0.795) interpretation of reliability *: poor (poor to good)	[N] [N]	0.583 (−0.228–0.890) interpretation of reliability *: moderate (poor to good)
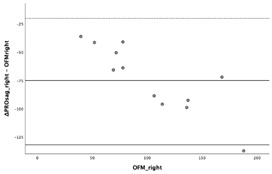
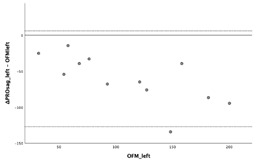
[N] [N]	0.428 (−0.176–0.821) interpretation of reliability *: poor (poor to good)	[N] [N]	0.653 (−0.230–0.909) interpretation of reliability *: moderate (poor to excellent)
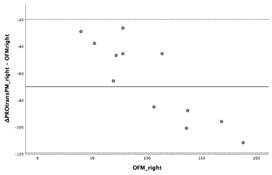
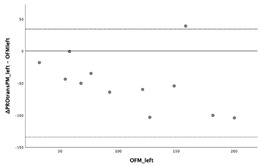
[N] [N]	0.433 (−0.144–0.826) interpretation of reliability *: poor (poor to good)	[N] [N]	0.626 (−0.263–0.899) interpretation of reliability *: moderate (poor to good)
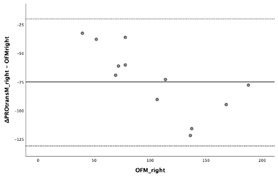
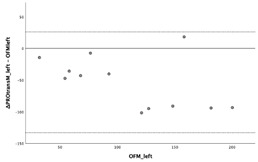
**(e) Edentulous, Complete Denture**			
Right side of the jaw	**ICC (95% CI)**	Left side of the jaw	**ICC (95% CI)**
[N] [N]	0.650 (0.268–0.824) interpretation of reliability *: moderate (poor to good)	[N] [N]	0.729 (0.434–0.864)interpretation of reliability *: moderate (poor to good)
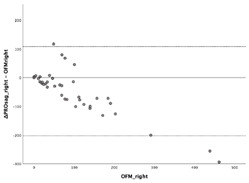
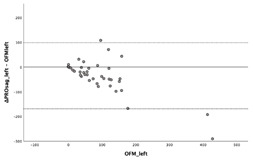
[N] [N]	0.693 (0.325–0.850) interpretation of reliability *: moderate (poor to good)	[N] [N]	0.733 (0.482–0.860) interpretation of reliability *: moderate (poor to good)
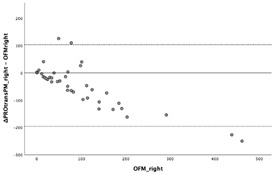
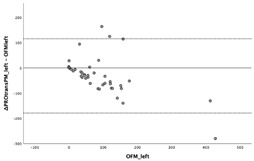
[N] [N]	0.740 (0.414–0.875) interpretation of reliability *: moderate (poor to good)	[N] [N]	0.809 (0.533–0.912) interpretation of reliability *: good (moderate to excellent)
